# Supplementary material for: Integrative Transcriptome, miRNAs, Degradome, and Phytohormone Analysis of Brassica rapa L. in Response to Plasmodiophora brassicae
Source: Int J Mol Sci. 2023 Jan 26;24(3):2414. doi: 10.3390/ijms24032414 (PMC9916777; doi:10.3390/ijms24032414)
Supplement: Supplementary file 1 [file ijms-24-02414-s001.zip › supplementary Table S1.pdf]

Table S1 Statistics of small RNA sequencing data output

sample	Raw read	Q 30(%)	Containing 'N' read R	Length < 18	Length > 30	Clean read
S-0d-1	18989492	97.61	69	4982667	1168895	12837861
S-0d-2	21981402	96.9	47	8140437	1060361	12780557
S-0d-3	20782377	96.31	29	7341797	1662314	11778237
S-3d-1	17487760	97.27	37	4859922	780262	11847539
S-3d-2	14971147	97.47	53	2955050	1187505	10828539
S-3d-3	13048923	97.28	48	1873000	1167351	10008524
S-9d-1	16065904	97.33	59	4839820	977868	10248157
S-9d-2	18951086	96.92	32	5983845	1057915	11909294
S-9d-3	17780071	97.32	47	5426652	1036765	11316607
S-20d-1	14494273	97.27	56	177677	495517	13821023
S-20d-2	12941257	97.6	38	1306386	210856	11423977
S-20d-3	12919070	97.04	31	286677	393097	12239265
R-0d-1	22798822	97.44	80	3857954	3701283	15239505
R-0d-2	20886549	97.3	62	3681544	2139338	15065605
R-0d-3	23551608	97.31	70	6532371	2577496	14441671
R-3d-1	25170621	96.61	158	3494079	2002362	19674022
R-3d-2	36631238	96.74	283	9617040	1376534	25637381
R-3d-3	15075627	96.65	129	587774	3234760	11252964
R-9d-1	20971555	96.61	148	1089336	4679063	15203008
R-9d-2	16166735	94.94	64	2859941	1883337	11423393
R-9d-3	18761498	96.6	87	3251017	2867736	12642658
R-20d-1	17683059	96.85	51	2032261	2626745	13024002
R-20d-2	16422681	97.49	47	2867285	1901296	11654053
R-20d-3	16426816	97.42	54	3451091	1119736	11855935
