# Supplementary figures and images for: Integrative Transcriptome, miRNAs, Degradome, and Phytohormone Analysis of Brassica rapa L. in Response to Plasmodiophora brassicae
Source: Int J Mol Sci. 2023 Jan 26;24(3):2414. doi: 10.3390/ijms24032414 (PMC9916777; doi:10.3390/ijms24032414)

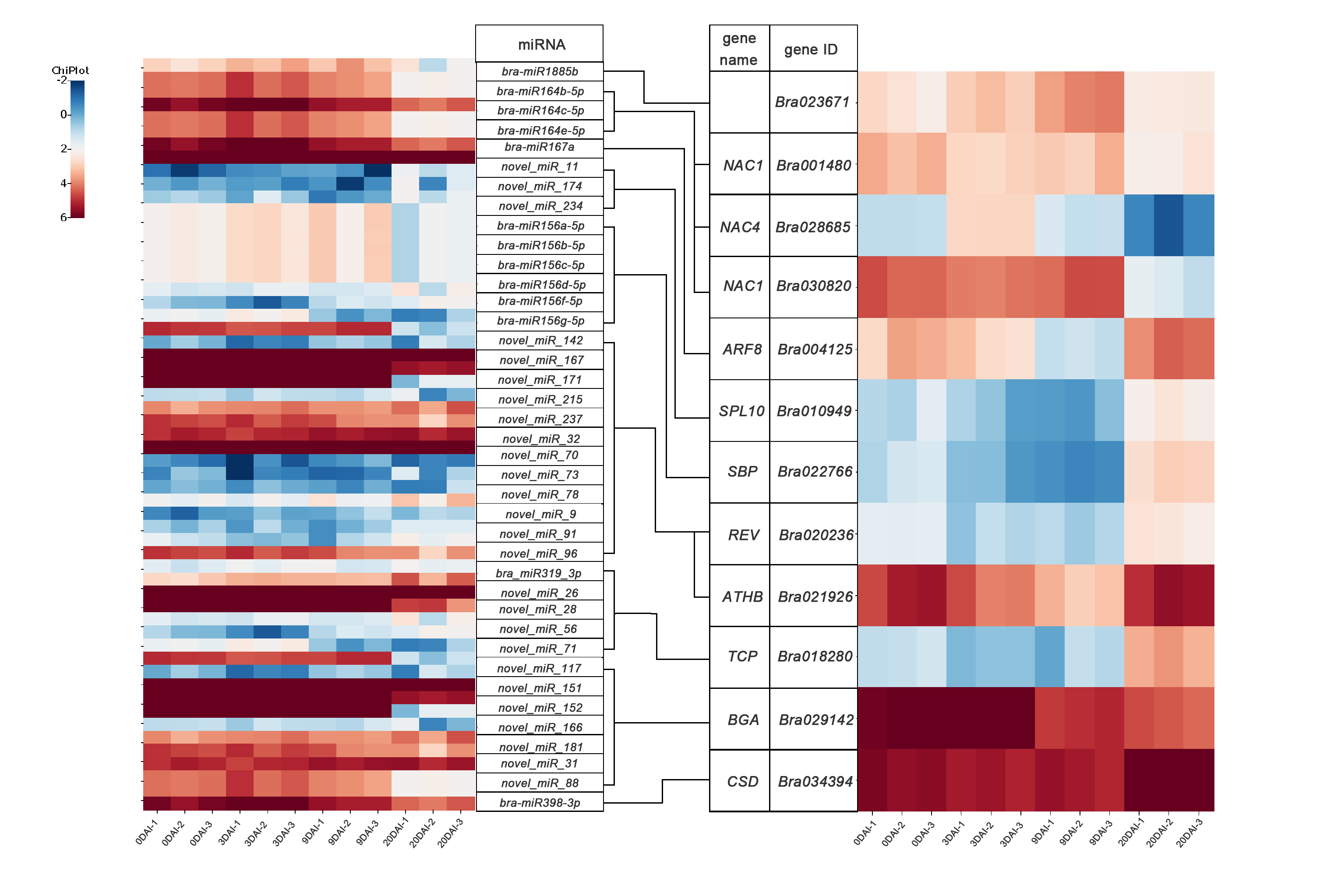

Supplement: Supplementary file 1 [file ijms-24-02414-s001.zip › supplementary Figure S1.png]

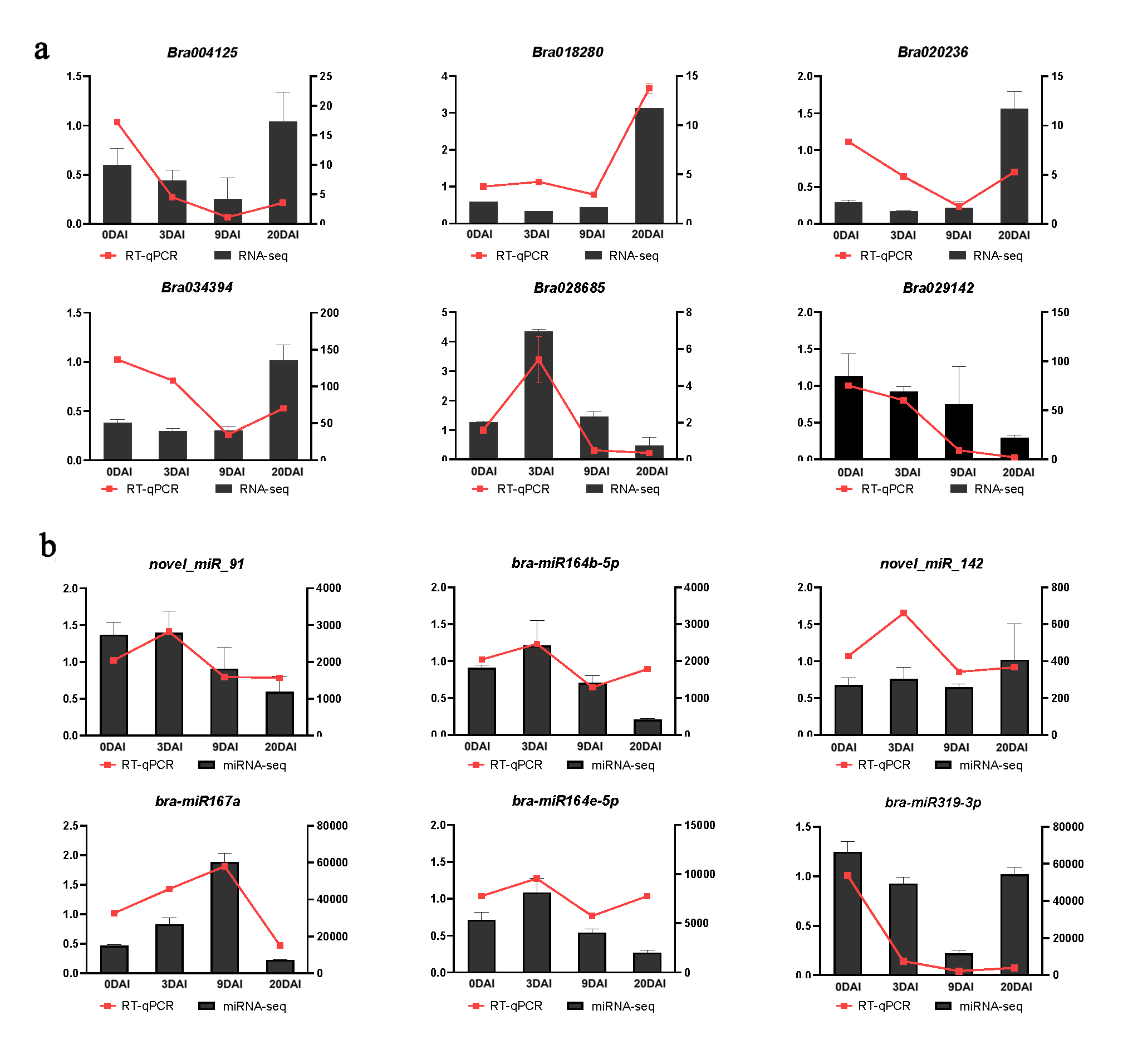

Supplement: Supplementary file 1 [file ijms-24-02414-s001.zip › supplementary Figure S2.png]
